# *O-fucosylation* of the Notch Ligand mDLL1 by POFUT1 Is Dispensable for Ligand Function

**DOI:** 10.1371/journal.pone.0088571

**Published:** 2014-02-12

**Authors:** Julia Müller, Nadia A. Rana, Katrin Serth, Shinako Kakuda, Robert S. Haltiwanger, Achim Gossler

**Affiliations:** 1 Institut für Molekularbiologie, Medizinische Hochschule Hannover, Hannover, Germany; 2 Department of Biochemistry and Cell Biology, Stony Brook University, Stony Brook, NY, United States of America; Heart Science Centre, Imperial College London, United Kingdom

## Abstract

Fucosylation of Epidermal Growth Factor-like (EGF) repeats by protein O-fucosyltransferase 1 (POFUT1 in vertebrates, OFUT1 in Drosophila) is pivotal for NOTCH function. In Drosophila OFUT1 also acts as chaperone for Notch independent from its enzymatic activity. NOTCH ligands are also substrates for POFUT1, but in Drosophila OFUT1 is not essential for ligand function. In vertebrates the significance of POFUT1 for ligand function and subcellular localization is unclear. Here, we analyze the importance of O-fucosylation and POFUT1 for the mouse NOTCH ligand Delta-like 1 (DLL1). We show by mass spectral glycoproteomic analyses that DLL1 is O-fucosylated at the consensus motif C^2^XXXX(S/T)C^3^ (where C^2^ and C^3^ are the second and third conserved cysteines within the EGF repeats) found in EGF repeats 3, 4, 7 and 8. A putative site with only three amino acids between the second cysteine and the hydroxy amino acid within EGF repeat 2 is not modified. DLL1 proteins with mutated O-fucosylation sites reach the cell surface and accumulate intracellularly. Likewise, in presomitic mesoderm cells of POFUT1 deficient embryos DLL1 is present on the cell surface, and in mouse embryonic fibroblasts lacking POFUT1 the same relative amount of overexpressed wild type DLL1 reaches the cell surface as in wild type embryonic fibroblasts. DLL1 expressed in POFUT1 mutant cells can activate NOTCH, indicating that POFUT1 is not required for DLL1 function as a Notch ligand.

## Introduction

The evolutionarily conserved Notch signaling pathway mediates direct cell-to-cell communication and regulates numerous developmental processes [1]–[5]. Notch genes encode transmembrane proteins that act at the surface of a cell as receptors for transmembrane proteins encoded by the *Delta* and *Serrate* (called Jagged (*Jag*) in mammals) genes. NOTCH as well as its ligands have a gene-specific number of epidermal growth factor-like (EGF) repeats in their extracellular domains [6]–[8] that are critical for receptor-ligand interaction. Upon ligand binding, the intracellular portion of NOTCH is proteolytically released, translocates to the nucleus, and by binding to a transcriptional regulator of the CSL family, activates transcription of target genes [9]–[15].

Posttranslational modification of NOTCH by O-fucose is essential for Notch signaling both in Drosophila and mammals [16], [17]. Protein O-fucosyltransferase 1 (POFUT1), which is encoded by Ofut1 in Drosophila and Pofut1 in mammals [18], adds O-fucose to Ser or Thr residues that are part of a consensus motif in certain EGF repeats of NOTCH [19], [20]. O-Fucose residues on EGF repeats can be further modified by Fringe (FNG) proteins, fucose-specific β1,3 N-acetylglucosaminyltransferases that act in the trans-Golgi [20]–[22]. Notch modification by Fringe affects the ability of ligands to activate Notch receptors in a context-dependent manner [23]–[25], but O-fucosylation was dispensable for Notch activity during embryonic neurogenesis in Drosophila [26].

In addition to providing the substrates for Fringe proteins, POFUT1 appears to influence Notch function in several ways. Analysis of OFUT1 mutants in Drosophila led to the conclusion that OFUT1 has a chaperone activity distinct from its fucosyltransferase activity that assists in Notch folding and cell-surface presentation [27], [28]. Another study suggested that Drosophila OFUT1 also acts extracellularly and regulates Notch endocytosis thereby maintaining stable Notch presentation at the cell surface [29]. In mammalian cells in culture and in haematopoietic cells in mice loss of POFUT1 did not prevent surface expression of Notch receptors but caused reduced ligand binding and Notch activity [30], [31], whereas in the paraxial mesoderm of mice lacking POFUT1 Notch1 was reported to accumulate in the ER [32]. These apparent differences notwithstanding, POFUT1 is clearly required for normal Notch function.

EGF repeats of the ligands also contain recognition sites for POFUT1 that are O-fucosylated [33]. OFUT1 appears to be dispensable for folding or function of ligands in Drosophila [16], but the significance of O-fucose modification or fucosyltransferase-independent functions of POFUT1 for the activity and localization of vertebrate ligands is unclear. Here, we focus on the murine Notch ligand DLL1. We show that EGF repeats 3, 4, 7, and 8 are stoichiometrically modified with O-fucose at the predicted consensus sites. DLL1 variants in which the Ser or Thr residues in the consensus sites were replaced with Ala and Val residues, respectively accumulated intracellularly in addition to their cell surface localization. However, in cells lacking POFUT1 stably overexpressed wild type DLL1 protein was presented at the cell surface at similar relative amounts as in wild type cells, and non-fucosylated DLL1 was able to effectively activate Notch.

## Materials and Methods

### Ethics statement

Animal experiments were performed according to the German rules and regulations (Tierschutzgesetz), and approved by the ethics committee of Lower Saxony for care and use of laboratory animals LAVES (Niedersächsisches Landesamt für Verbraucherschutz und Lebensmittelsicherheit). Mice were housed in the central animal facility of Hannover Medical School (ZTL) and were maintained as approved by the responsible Vetinary Officer of the City of Hannover. Animal welfare was supervised and approved by the Institutional Animal Welfare Officer (Tierschutzbeauftragter). For embryo collection mice were sacrificed by cervical dislocation.

### Generation of expression constructs

The DLL1-FLAG protein variants carrying mutations in one/multiple fucosylation sites were generated using the “QuikChange Site-directed Mutagenesis Kit” (Stratagene) according the manufacturer's protocol and a pTracer-DLL1Flag plasmid [34] as a template. Mutations were designed to convert Ser to Ala, or Thr to Val, and confirmed by DNA sequencing. Mutated cDNAs were used as templates for subsequent mutageneses. Primers used for site-directed mutagenesis:
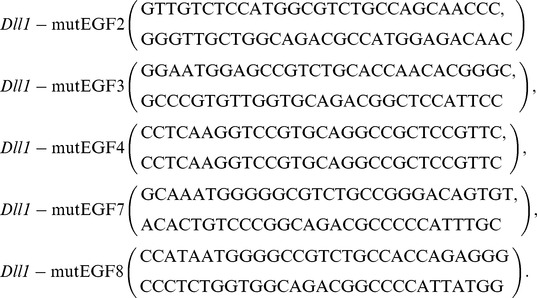
Cell lines

Dll1 expression constructs were introduced into CHO cells by transfection using JetPei (BIOMOL) according to the manufacturer's instruction followed by Neomycin and Zeocin (Invitrogen) selection. Each stable cell line was subcloned by limiting dilution. Cells were plated at a density of 0.5 cell/well in 96-well-plates and monoclonal cultures were expanded. HeLaN1 cells have been described [11]. Primary mouse fibroblasts (MEFs) were prepared from E9.5 POFUT1+/+ and POFUT1−/− embryos [17], and immortalized with pRRL.PPT.SF.largeT.pre which contains SV40T cloned into pRRL.PPT.SF.GFP.pre [35]. Fibroblasts stably expressing DLL1 were obtained by transfection of these MEFs with a wild type Dll1-Flag construct.

### Immunohistochemistry

Immunocytochemistry and whole-mount immunohistochemistry was performed as described [34] and visualized using a laser scanning microscope (Zeiss LSM 510 Meta connected to an Axiovert 200 M).

### Antibodies

Primary antibodies were: DLL1 (rabbit, Santa Cruz, sC-9102), Alpha 1 Sodium Potassium ATPase (mouse, Abcam, ab7671), GM130 (mouse, BD Biosciences, 610823), KDEL (mouse, Abcam, ab1223), EEA1 (rabbit, Abcam, ab2900), Rab5 (mouse, Sigma, R7904), Rab6A (mouse, Sigma, WH0005870M1), Transferrin receptor (mouse, Zymed, 13-6800), Caveolin (rabbit, BD Biosciences, 610060), Rab11 (rabbit, Sigma, R5903), Pan-Cadherin (mouse, Sigma, C1821), β-Actin (mouse, MPBiomedicals, 69100). Monoclonal antibodies against DLL1 were described [34]. Secondary antibodies were: Donkey anti-rat-Alexa488, goat anti-rat-Alexa488, goat anti-mouse-Alexa555, goat anti-rabbit-Alexa488, goat anti-mouse-Alexa633, goat anti-rabbit-Alexa555 (Molecular probes, Invitrogen).

### Surface biotinylation and Quantification of DLL1 protein

For non-quantitative analyses cell surface biotinylation and subsequent streptavidin-precipitation was performed as described previously [34]. After cell lysis the samples were divided into equal parts for immunoprecipitation with either Flag-Agarose Beads (M2, Sigma) to detect DLL1 irrespective of its cellular localization and Streptavidin-Sepharose (GE) to detect the surface biotinylated fraction. For quantitative analyses 0,5×10^6^ mouse fibroblast cells were plated on 6-cm dishes and biotinylated as described [34]. After cell lysis a 50 µl aliquot was taken and mixed with 2 x sample buffer. The remaining lysate was used for immunoprecipitation (IP) with NeutrAvidin Agarose Resin (Thermo Scientific, 29200) over night at 4°C. The resin was washed three times in Ripa lysis buffer (50 mM Tris/HCl, pH 7.6, 150 mM NaCl, 1 mM EDTA, pH 8.0, 1% TritonX100, 0.25% DOC and 0.1% SDS supplemented with Protease Inhibitor Cocktail tablets (Roche, 04693159)) and resuspended in 2 x sample buffer. The supernatant of the IP was subjected to a second round of IP to confirm that DLL1 was quantitatively precipitated in the first IP. Protein from whole cell lysates and immunoprecipitated proteins were subjected to SDS-PAGE and analyzed by Western blotting using the Fuji LAS-4000 Western blot detection system. Protein bands were quantitated using ImageJ [36].

### Notch Transactivation analysis

NOTCH1 expressing HeLa cells (HeLaN1) were co-cultured with POFUT1+/+ and POFUT1−/− fibroblasts expressing wild type DLL1, untransfected CHO cells and CHO cells expressing wild type DLL1and DLL1 protein variants (I-IV). Activation of NOTCH1 was detected in the lysates by Western-Blot analysis with the anti-Cleaved Notch1 (Val1744) antibody (rabbit, Cell Signaling 2421) which detects the Notch1 intracellular domain (NICD) only after activation and S3 cleavage.

### Mice

POFUT1 mutant mice were described [17].

### Glycoproteomic analysis of mouse DLL1

A plasmid encoding the extracellular domain of mouse DLL1 (amino acids 1–535) fused to the Fc portion of human immunoglobulin (pCDM8-DLL1-Fc) was transiently transfected into HEK-293 T cells as described [37]. Four hours after transfection, cells were washed with PBS and grown in EX-CELL 293 HEK serum-free medium (SAFC Biosciences) for four days. Protein was purified from the medium using Protein A-Sepharose. Purified protein was reduced, alkylated and subjected to in-gel digestion with trypsin, chymotrypsin or V8 protease as described [38] The resulting peptides were analyzed by nanoLC-MS/MS using an Agilent nanoHPLC-CHIP system coupled to a model 6430 Ion Trap mass spectrometer as described [38]. O-Fucosylated peptides were identified by neutral loss searches, and semi-quantitative Extracted Ion Chromatograms of selected ions were generated to compare relative amounts of O-fucosylated and unfucosylated forms of each peptide [38]. Raw data for the chromatograms was processed with a Gaussian smoothing algorithm provided with the Data Analysis software from Agilent.

## Results

### EGF repeats 3, 4, 7, and 8 of DLL1 are O-fucosylated

**Figure 1:**
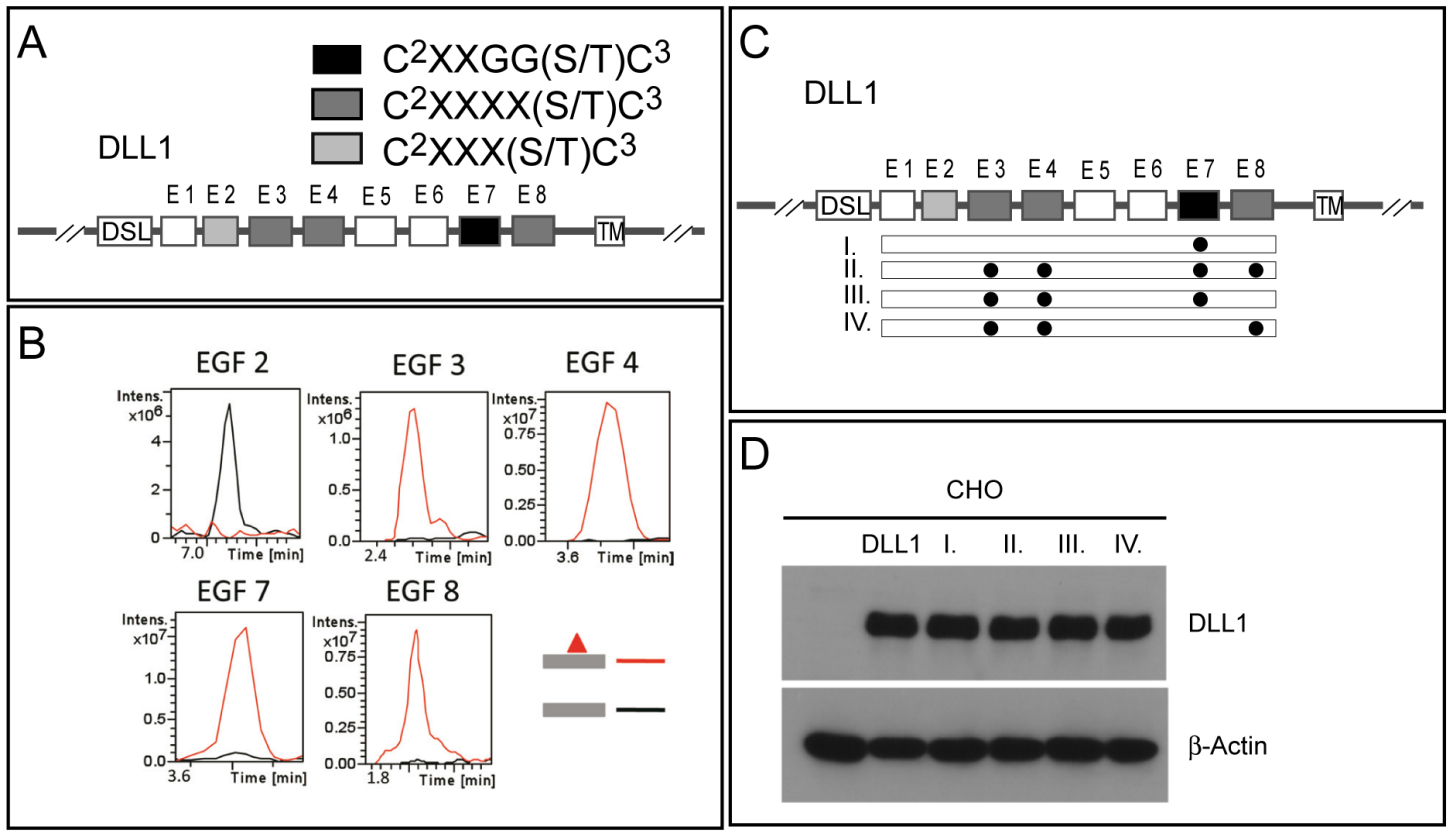
Schematic overview of DLL1 wild type and mutant proteins and mass spectrometry results. (A) Schematic representation of the DLL1 protein. Boxes indicate DSL, EGF1-EGF8 (E1–E8) repeats, and the transmembrane (TM) domain. The presence of different classes of O-fucosylation consensus sites is indicated by light grey, dark grey and black shading. (B) Extracted Ion Chromatograms (EIC) show that peptides derived from EGF repeats 3, 4, 7, and 8 are modified at high stoichiometry, but the peptide derived from EGF repeat 2 is not O-fucosylated. Masses corresponding to the glycopeptides (red lines) or unmodified peptide (black lines) used to generate the EIC are shown in Figure S1. (C) Schematic overview over single and multiple mutations (indicated by black dots aligned to EGF domains) introduced into EGF repeats of DLL1. (D) Western Blot analysis of cell lysates of clonal CHO cell lines stably expressing DLL1 and mutant variants at comparable levels. Beside CHO control cells (CHO), and CHO cells expressing DLL1wt (DLL1), roman letters refer to DLL1mEGF7 (I), DLL1mEGF3/4/7/8 (II), DLL1mEGF3/4/7 (III) and DLL1mEGF3/4/8 (IV).

The original consensus sequence for O-fucose modifications of Ser or Thr residues in EGF repeats was C^2^XXGG(S/T)C^3^, where C^2^ and C^3^ are the second and third conserved cysteines of an EGF repeat and X is any amino acid [39]. Further studies of Notch have identified a broader consensus sequence C^2^XXXX(S/T)C^3^ [20]. Mouse DLL1 contains one narrow C^2^XXGG(S/T)C^3^ site in EGF repeat 7, and three broader C^2^XXXX(S/T)C^3^ sites in EGF repeats 3, 4, and 8 (Fig. 1 A). EGF repeat 2 contains an additional putative consensus site, C^2^XXX(S/T)C^3^, with only 3 amino acids between the second cysteine and the hydroxy amino acid. Although such sites have been predicted to be modified [33], no evidence for their O-fucosylation has yet been provided. A similar site in EGF repeat 15 of NOTCH1 is not O-fucosylated [20].

To identify the site(s) in DLL1 that are O-fucosylated, a plasmid encoding a fusion protein with the extracellular domain of mouse DLL1 fused to the Fc portion of human Ig was transiently transfected into HEK293T cells, and the resulting DLL1-Fc protein was purified from the medium. The purified protein was subjected to in-gel protease digestion, and the resulting peptides were analyzed by nano-LC-MS/MS to identify O-fucosylated peptides. Fucosylated peptides from EGF repeats 3, 4, 7, and 8 containing the appropriate consensus sequence were identified (Table 1, Figure 1B and Figure S1). Semiquantitative analysis reveals that each of the sites is modified at very high stoichiometry (Figure 1B), suggesting that the fucosylation machinery is highly efficient. The peptide from EGF repeat 2 containing the potential O-fucosylation site was not modified (Table 1, Figure 1C), confirming that the consensus site requires at least 4 amino acids between the second cysteine and the modified residue.

**Table 1:** Peptides from mouse DLL1 identified with O-fucose modifications.

EGF	Sequence	Parent Ion (M+H^−^)	Deglyco Product (M+H^−^)	Mass Ä	Predicted Mass (M+H^−^)
2	^256^CIRYPGCLHGTCQQPWQCNCQE^277^	2836.3*	2836.3	0.0	2854.2
3	^299^NGATCTNTGQGSYTCSCR^316^	2141.0	1995.0	146.0	1996.1
4	^331^CAPSPCKNGASCTDLE^346^	1913.2	1767.2	146.0	1767.9
7	^438^YCEDNVDDCASSPCANGGTCR^458^	2556.1	2409.1	147.0	2409.5
8	^486^HAPCHNGATCHQRGQRYMCE^505^	2617.8	2472.2	145.6	2471.7

O-fucosylated peptides were identified by neutral loss of mass corresponding to fucose (146 daltons, see Figure S1) upon fragmentation. Spectra for each glycopeptide identified here are shown in the Figure S1. All masses were converted to the equivalent of singly charged (M+H^+^) for the table. For each glycopeptide, the mass of the parent ion, the deglycosylated product (lacking fucose), and the difference between these (corresponding to the mass of the modification) is shown. The predicted mass of the unglycosylated peptide is also shown. All peptide masses are adjusted for carbamidomethylation of cysteines. For peptides with a mass below 2000 Da, monoisotopic masses were used. For those above 2000 Da, average masses were used. Predicted O-fucose modification sites are bold underlined, and cysteines within the consensus sequence, C^2^XXXX(S/T)C^3^ are bold. *Note that this ion lost a water (16 Daltons).

### Mutations disrupting O-fucosylation sites cause intracellular accumulation of DLL1

**Figure 2:**
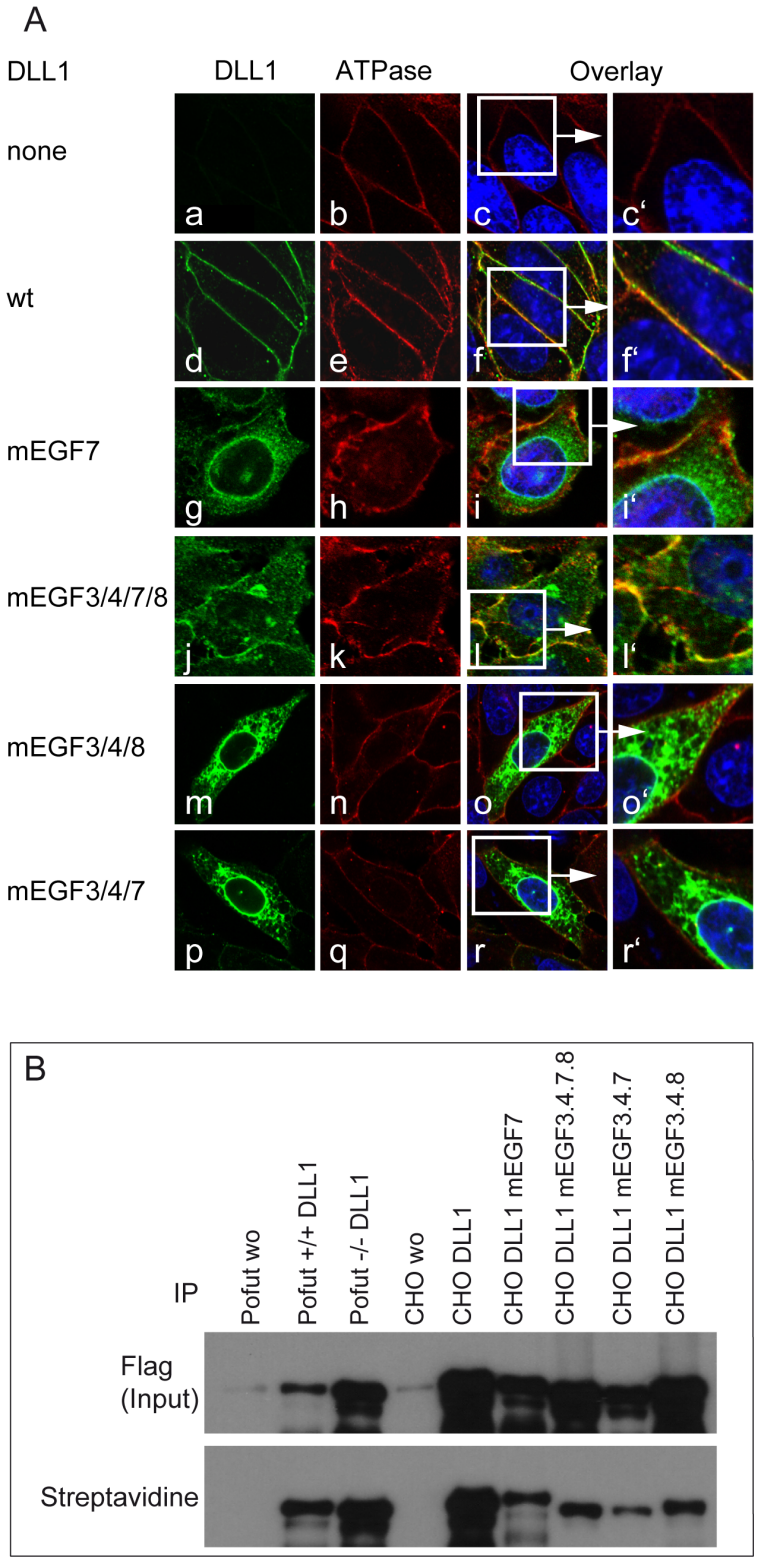
Subcellular localization of DLL1 protein variants in CHO cells. (A) Confocal images stained with antibodies against DLL1 and a marker for the cell surface (Sodium potassium ATPase). (a-r) CHO cells stably transfected with control (a-c), wild type DLL1 (d-f), or mutant DLL1 (g-r) show wild type DLL1 on the surface (d-f′) and the protein variants DLL1mEGF7, DLL1mEGF3/4/7/8, DLL1mEGF3/4/7 and DLL1mEGF3/4/8 were detected on the cell surface and intracellularly (g-i′, j-l′, m-o′, p-r′). (B) Western Blot analysis of DLL1 protein variants after cell surface biotinylation, cell lysis and immunoprecipitation with Flag-Agarose beads (Flag Input) and Streptavidin-Sepharose to detect the cell surface biotinylated fraction (Streptavidin). Cells and constructs are indicated above the lanes.

**Figure 3:**
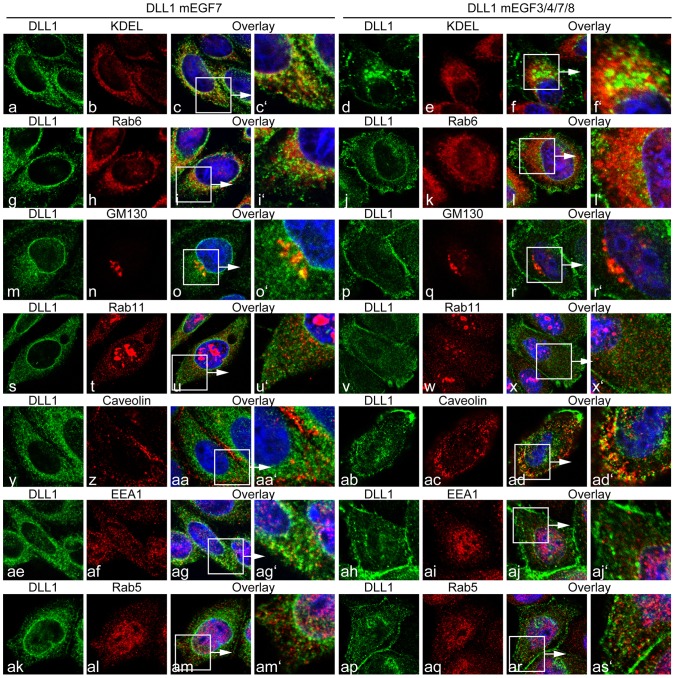
Colocalization of DLL1mEGF7 and DLL1mEGF3/4/7/8 protein with intracellular compartment markers. Confocal images of cells stably expressing DLL1mEGF7 or DLL1mEGF3/4/7/8 co-stained with antibodies against DLL1 and the ER markers KDEL (a-f′) and Rab6A (g-l′), the trans-Golgi markers GM130 (m-r′) and Rab11 (s-x′), the endocytotic vesicle marker Caveolin (y-Ad′), and the early endosome markers EEA1 (Ae-Aj′) and Rab5 (Ak-As′). DLL1mEGF7 is present on the cell surface and intracellulary partially overlaping with KDEL, Rab6A and GM130, and to a lesser extent with Rab11, Caveolin, EEA1 and Rab5. The DLL1mEGF3/4/7/8 variant shows a similar distribution.

To address the significance of these potential O-fucosylation sites for DLL1 function we generated DLL1 variants (Figure 1 C), in which the Ser or Thr residues in the consensus sites were replaced with Ala and Val residues, respectively, to prevent O-fucosylation, and established CHO cell lines expressing these variants at similar levels (Figure 1 D). First, we analyzed the subcellular localization of these variants by indirect immunofluorescence and surface biotinylation. Wild type DLL1 was detected predominantly on the cell surface overlapping with Sodium/Potassium ATPase (Figure 2A f-f′), an ion-channel located in the plasma membrane [40], and was efficiently labelled by surface biotinylation (Figure 2B). In contrast, a DLL1 variant in which all O-fucosylation sites were mutated (DLL1mEGF3/4/7/8; in Fig. 1 C) accumulated intracellularly in the perinuclear region in addition to localization on the cell surface (Figure 2A l-l′, and Figure 2B). Also a DLL1 variant, in which only the narrow O-fucosylation site in EGF7 was mutated (mEGF7) was present at the plasma membrane (Figure 2A i-i′, and Figure 2B), in addition to strong intracellular staining. Likewise a variant with mutated fucosylation sites in EGF repeats 3, 4 and 8 (DLL1mEGF3/4/8; in Fig. 1 C) showed significant intracellular staining in addition to its localization at the cell membrane (Figure 2A o-o′, and Figure 2B). Collectively, these results raised the possibility that the efficient presentation at the cell surface requires modification of DLL1 by O-fucose and O-fucosylation at multiple sites, or that the amino acid changes introduced by mutation cause misfolding and intracellular retention of DLL1. To test whether the mutation of *O*-fucosylation sites leads to the accumulation of DLL1 in the endoplasmatic reticulum (ER), the Golgi apparatus, or endosomes we co-stained CHO cells expressing DLL1mEGF7 or DLL1mEGF3/4/7/8 for DLL1 and markers for these subcellular compartments (Figure 3). Both DLL1mEGF7 and DLL1mEGF3/4/7/8 showed some colocalization with markers for the ER (KEDL, Rab6A), the Golgi (GM130, Rab11) and endosomes (EEA1, Rab5, Caveolin). However, there was also substantial punctate DLL1 staining in the cytoplasm that did not overlap with these markers indicating that DLL1 is not predominantly associated with one particular of the analyzed compartments.

### O-fucosylation is not essential for cell surface localization of DLL1 in vivo and in vitro

**Figure 4:**
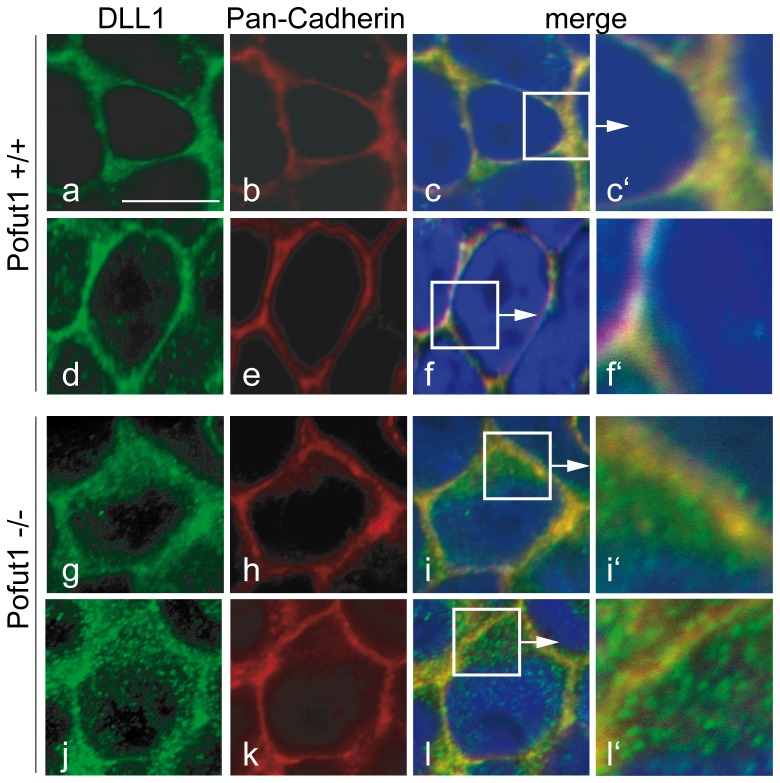
Localization of endogenous DLL1 in PSM cells lacking POFUT1. Confocal images of flat mounted PSMs from wild type and POFUT1 mutant E9.5 embryos (a-f). In wild type PSM cells, DLL1 was present almost exclusively on the cell surface and colocalized with Pan-Cadherin staining (c-c′). In POFUT1 mutant PSM cells most of the DLL1 protein was detected intracellularly in punctae or dots, reflecting significantly reduced colocalization with Pan-Cadherin (f-f′).

Overexpression or the altered amino acid sequence of O-fucosylation sites rather than the lack of O-fucosylation might contribute to the abnormal localization of DLL1mEGFs in CHO cells. To test the apparent requirement for O-fucosylation independently, we analyzed DLL1 in whole mount stained presomitic mesoderms (PSMs) of POFUT1 null mutant embryos [17]. In wild type PSM cells, DLL1 significantly colocalized with Pan-Cadherin (PanCAD) staining (Fig. 4 c-c′, f-f′) indicating cell surface localization as reported previously [34]. In addition, DLL1 was present in cytoplasmic punctae. Likewise, in POFUT1 mutant PSM cells DLL1 was clearly present at the cell surface (Fig. 4 i-i′, l-l′), in addition to significant cytoplasmic staining. Thus, also under physiological conditions, POFUT1 is not essential for cell surface presentation of DLL1.

**Figure 5:**
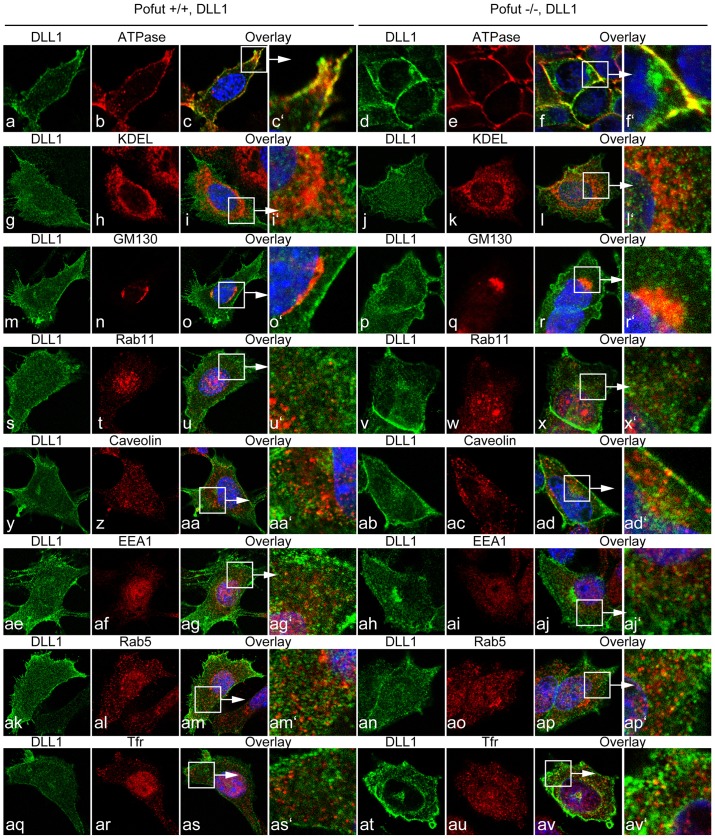
Localization of endogenous DLL1 in wild type and POFUT1 mutant MEFs. Confocal images of wt and Pofut deficient cells stably overexpressing flag-tagged DLL1. Costaining were performed with antibodies against DLL1 (green) and marker for intracellular compartments (red). Both in wt and POFUT1 deficient cells DLL1 is located on the cell surface and colocalizes with the cell surface marker ATPase (a-f′). In addition, DLL1 protein was also detected intracellulary in both cell lines showing partially colocalization with the ER markers KDEL (g-l′), the trans-Golgi markers GM130 (m-r′) and Rab11 (s-x′), the endocytotic marker Caveolin (y-ad), the early endosome markers EEA1 (ae-aj′), Rab5 (ak-ap′) and the transferrin receptor (TfR, aq-av).

To further analyze and quantitate a potential effect of O-fucosylation on DLL1 we immortalized fibroblasts (MEFs) from Pofut1^tm1Pst/tm1Pst^ null mutant [17] and wild type embryos, and established clonal cell lines stably overexpressing wild type DLL1. In wild type MEFs DLL1 staining localized to the cell surface overlapping with ATPase staining in addition to punctate intracellular staining (Figure 5 c-c′), Likewise, MEFs lacking POFUT1 showed DLL1 staining overlapping with ATPase staining at the cell surface, and strong intracellular DLL1 throughout the cytoplasm (Figure 5 f-f′). In an attempt to identify the intracellular compartment in which DLL1 might accumulate in the absence of POFUT1 we analyzed wild type and POFUT1 mutant MEFs expressing wild type DLL1 with ER, Golgi and endosome markers used for the analysis of CHO cells expressing mutant DLL1 proteins (Figure 5). Similar to the mutant DLL1 proteins wild type DLL1 colocalized to varying extents with markers for the ER, the Golgi and endosomes both in wild type and POFUT1 mutant MEFS. However, there was also substantial DLL1 staining in the cytoplasm that did not overlap with these markers and a significant enrichment of DLL1 in POFUT1 mutant cells was not detected in any of these compartments.

**Figure 6:**
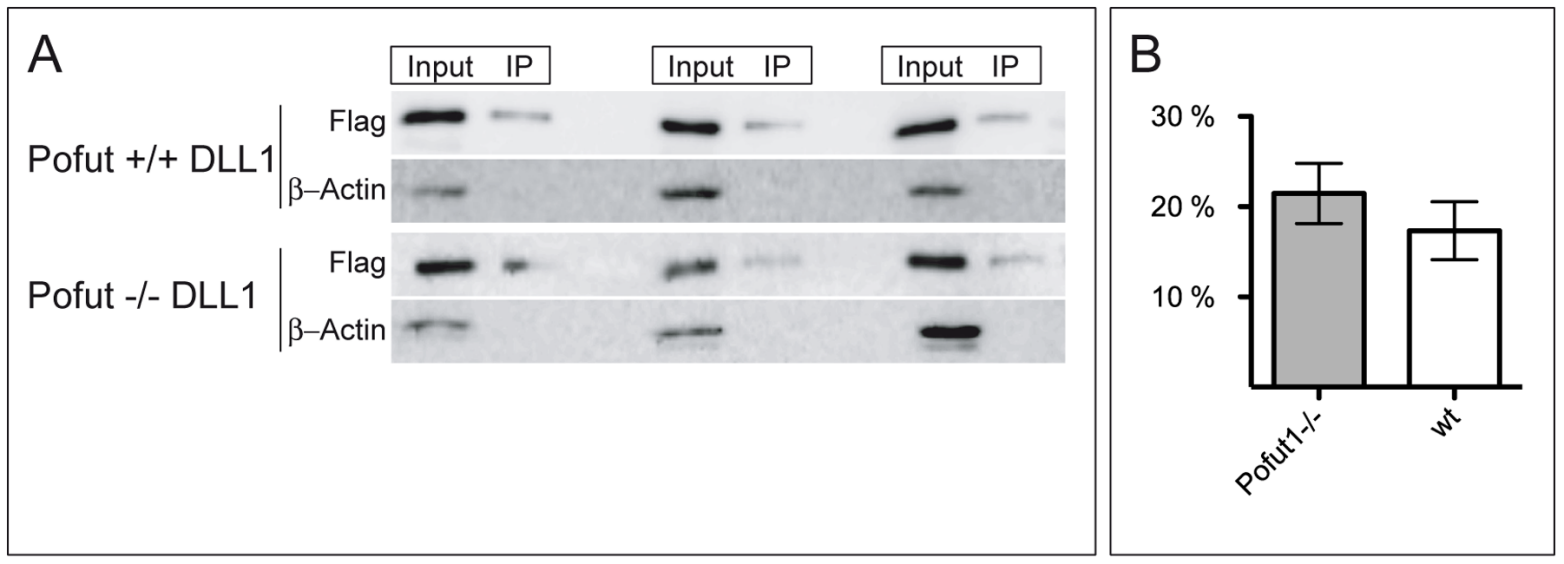
Analysis of DLL1 cell surface presentation by biotinylation. (A) Representative examples of Western blots used for quantification of DLL1 cell surface presentation in wt and POFUT1 deficient cells stably expressing flag-tagged DLL1. Input lane represents total DLL1 protein from whole cell lysates. IP lane represents DLL1 surface protein, which was biotinylated and immunoprecipitated with Neutravidin Agarose Resin (IP). As an additional control blots were reprobed with β-Actin antibody to exclude cell lysis during the biotinylation step. (B) Calculated relative cell surface levels of DLL1. 17.3% of total DLL1 protein was detected on the cell surface in POFUT wt cells, whereas in POFUT deficient cells 21.5% (p = 0.39) of total DLL1 reached the cell surface.

Consistent with the immunofluorescence data, DLL1 variants with mutated O-fucosylation sites expressed in CHO cells was detected at varying levels at the cell surface by surface biotinylation (Figure 2B). Likewise, wild type DLL1 expressed in POFUT1 mutant MEFS was detected at the cell surface by surface biotinylation (Figure 2B). To assess the portion of total DLL1 that is present on the cell surface in wild type and POFUT1 mutant cells quantitatively, we analyzed total and cell-surface DLL1 after surface biotinylation in six independent experiments. 17.3 and 21.5% of DLL1 was present on the surface of wild type and POFUT1 mutant MEFs, respectively (Figure 6 A, B), the difference being statistically not significant (p = 0.39), indicating that DLL1 expressed in MEFs reaches the cell surface efficiently even in the absence of POFUT1 and O-fucosylation.

### Unfucosylated DLL1 can activate Notch

**Figure 7:**
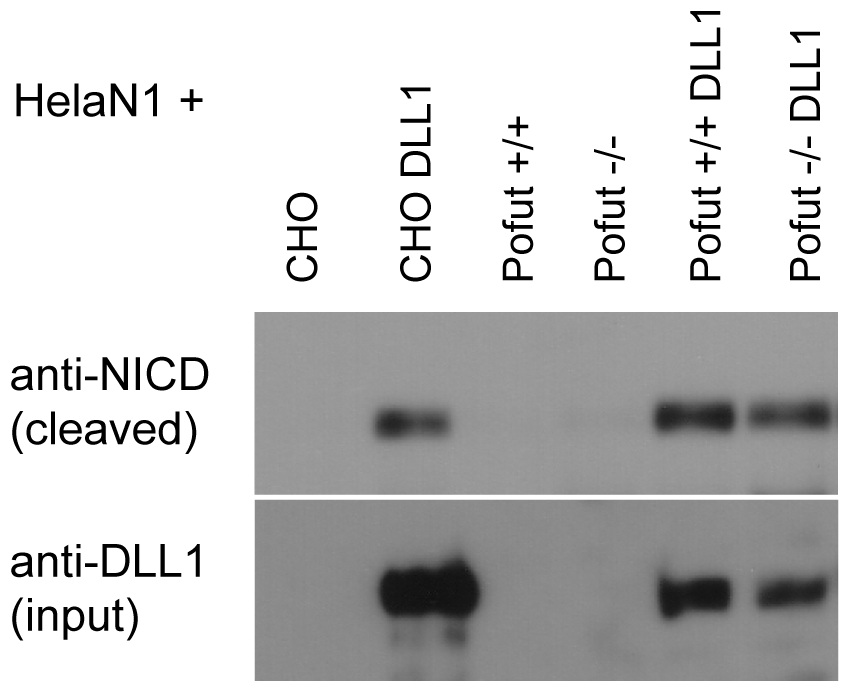
Notch activation by DLL1 in POFUT1 deficient fibroblasts. Western Blot analysis of cell lysates of NOTCH1 expressing HeLa cells (HeLaN1) co-cultured with POFUT1+/+ or POFUT1−/− fibroblasts or with CHO cells over-expressing DLL1 using the anti-Cleaved Notch1 (Val1744) antibody, which specifically detects the Notch1 intracellular domain (NICD) after S3 cleavage. Non-fucosylated DLL1 can efficiently activate Notch1.

To test whether O-fucosylation is important for DLL1-mediated activation of Notch, wild type and POFUT1 deficient cells expressing wt DLL1 were cocultured with HeLa cells stably expressing NOTCH1 (HeLaN1) and activated Notch was determined by Western Blot analysis using antibodies specifically recognizing NICD after S3 cleavage. DLL1 expressed on the surface of POFUT1 −/− cells induced S3 cleavage similar to wild type cells (Figure 7), indicating that O-fucosylation at EGF like repeats in the extracellular domain of DLL1 is not essential for DLL1 interaction with NOTCH1 and its activation in vitro.

## Discussion

Previous analyses had shown that rat Delta1 is O-fucosylated, but the extend and precise localization of this modification had not been determined (Panin et al., 2002).

Our mass spectrometry analysis has shown that EGF repeats in mouse DLL1 that contain the narrow C^2^XXGG(S/T)C^3^ (EGF 7) and the broader C^2^XXXX(S/T)C^3^ (EGF 3, 4, and 8) consensus sequence for O-fucosylation are stoichiometrically modified with O-fucose. In contrast, O-fucosylation was not detected on EGF 2, which only has three amino acids between the second cysteine and the hydroxy amino acid (C^2^XXX(S/T)C^3^). These findings corroborate results from the analyses of mouse NOTCH1, which indicated that both the narrow C^2^XXGG(S/T)C^3^ and the broader C^2^XXXX(S/T)C^3^ consensus sites are modified, while sites with three amino acids between C^2^ and the S/T residue are not [20].

The analysis by indirect immunofluorescence of DLL1 variants with mutated S/T residues in the fucosylation consensus sites suggested that the mutant proteins accumulated intracellularly. Similarly, in cells lacking POFUT1 cultured in vitro and in PSM cells lacking POFUT1 in vivo, we observed significant staining of intracellular DLL1 protein in addition to cell surface localization. The apparent retention of wild type DLL1 in the cytoplasm of POFUT1 mutant cells appeared less dramatic than that seen with mutant DLL1 proteins in wt cells, suggesting that variant proteins might not fold properly due to the introduced mutations. Thus, the exchange of the respective serine or threonine residue accepting O-fucose rather than the actual loss of O-fucosylation likely affect DLL1 localization in these cases. This idea is supported by results obtained from the analysis of Cripto, a coreceptor for Nodal, which is also O-fucosylated [41]: in Cripto threonine 72, the residue that is O-fucosylated, is of critical importance but not O-fucosylation, because the exchange of threonine 72 in Cripto with serine abolished Cripto function but not O-fucosylation [42].

The immunofluorescence data suggested an intracellular accumulation of non-fucosylated DLL1. However, quantitative analyses of surface-biotinylated DLL1 showed that there is no difference in the portion of DLL1 that is present at the cell membrane between wild type and POFUT1 mutant cells. These conflicting results are reminiscent of divergent observations concerning the requirement of POFUT1 for the localization of the mouse Notch1 protein. NOTCH1 detected by immunofluorescence accumulated intracellularly in PSM cells of mouse embryos lacking POFUT1 [32]. In contrast, quantitative analyses of NOTCH receptors indicated that they were present at wild type levels on the surface of Pofut1 mutant ES cells [30], and NOTCH receptor levels on the surface of POFUT1 deficient haematopoietic cells was only mildly reduced [31]. The reasons for these conflicting results are currently unclear, but may reflect cell type-specific requirements for POFUT1 or reside in methodological differences.

In contrast to NOTCH, Drosophila Delta and Serrate do not require OFUT1; clones of cells lacking OFUT1 efficiently activated NOTCH in adjacent wild type cells, indicating that loss of Drosophila OFUT1 had no obvious effect in signal sending cells and that ligands are folded and function normally [16]. Similarly, O-fucosylation of EGF-like repeats in mouse DLL1 does not appear to be of critical importance for binding to and activation of NOTCH, at least in co-culture assays in vitro. Hence, like Drosophila Delta mouse Delta1 does not require O-fucosylation for its surface presentation and activation of Notch.

## Supporting Information

Figure S1 **Identification of O-fucosylated peptides from EGF repeats 3, 4, 7, and 8 of mouse Dll1 by LC-MS/MS.** O-Fucosylated peptides were identified by searching for ions that lose a mass equivalent to fucose (146 daltons) upon collision induced dissociation (CID) fragmentation (Rana et al (2011) JBC 286: 31623–31637). For each peptide, an MS spectrum showing the selection of the parent ion for fragmentation (top) and an MS/MS spectrum showing the resulting CID fragmentation (bottom) is shown. Ions representing peptides are indicated by grey rectangles, and fucosylated peptides by grey rectangles with red triangles. Predicted masses of singly charged ions are shown in Table 1. Ions in the MS/MS spectrum corresponding to unmodified peptide (grey rectangles), as well as b- and/or y-ions from fragmentation of the peptide are shown. The sequence of the peptide is provided at the top of each spectrum. Note that the peptide from EGF2 is not fucosylated. The m/z for each of the fucosylated peptides (top panels) or unmodified peptide (bottom panel, except for EGF2) was used to generate the EIC figures shown in Figure 1B.
(PDF)
