# Supplementary figures and images for: O-fucosylation of the Notch Ligand mDLL1 by POFUT1 Is Dispensable for Ligand Function
Source: PLoS One. 2014 Feb 12;9(2):e88571. doi: 10.1371/journal.pone.0088571 (PMC3922938; doi:10.1371/journal.pone.0088571)

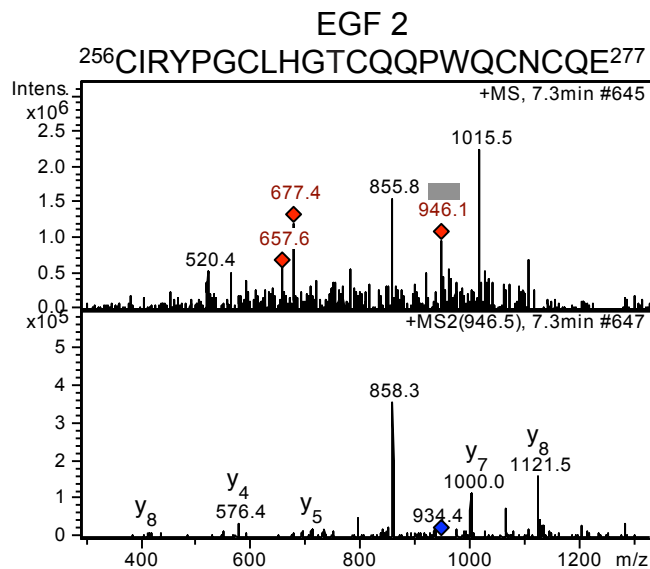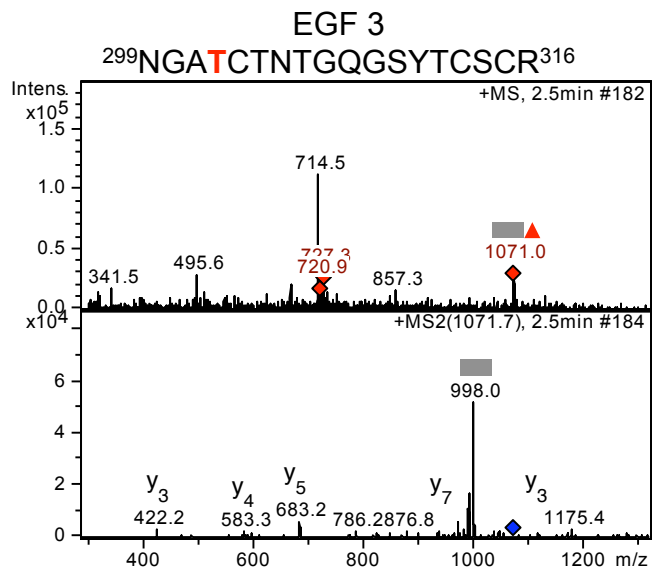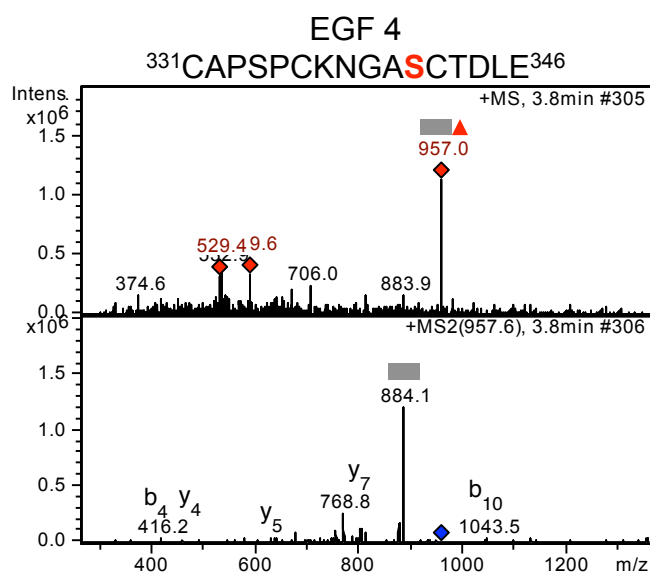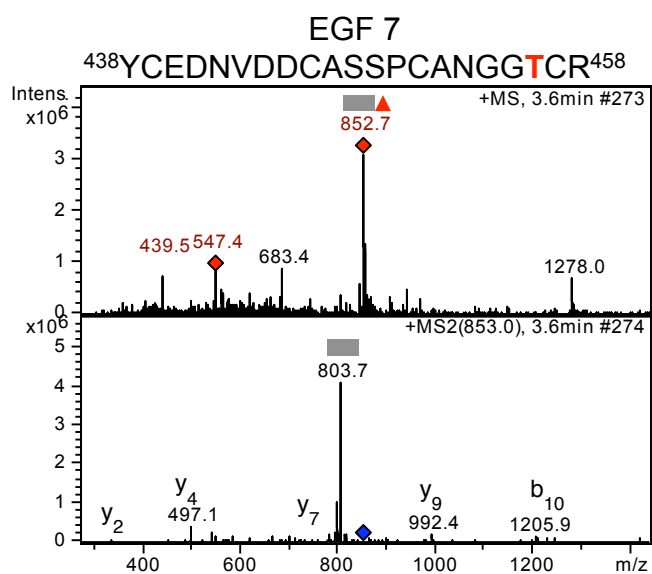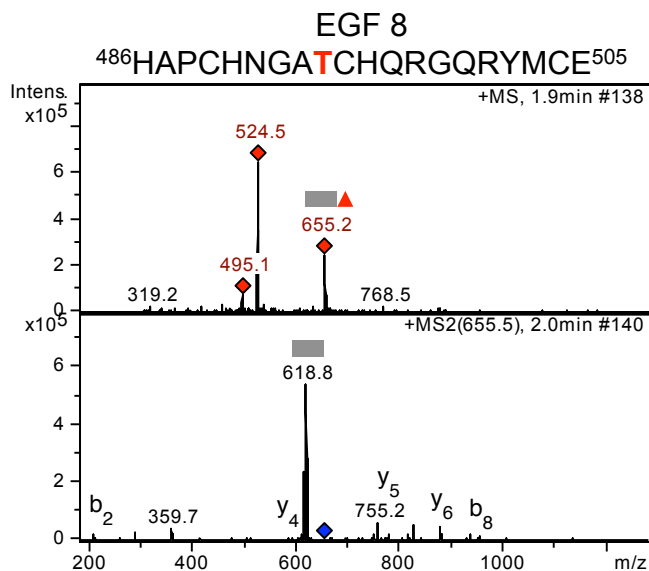

Supplement: Figure S1 — Identification of O-fucosylated peptides from EGF repeats 3, 4, 7, and 8 of mouse Dll1 by LC-MS/MS. O-Fucosylated peptides were identified by searching for ions that lose a mass equivalent to fucose (146 daltons) upon collision induced dissociation (CID) fragmentation (Rana et al (2011) JBC 286: 31623–31637). For each peptide, an MS spectrum showing the selection of the parent ion for fragmentation (top) and an MS/MS spectrum showing the resulting CID fragmentation (bottom) is shown. Ions representing peptides are indicated by grey rectangles, and fucosylated peptides by grey rectangles with red triangles. Predicted masses of singly charged ions are shown in Table 1. Ions in the MS/MS spectrum corresponding to unmodified peptide (grey rectangles), as well as b- and/or y-ions from fragmentation of the peptide are shown. The sequence of the peptide is provided at the top of each spectrum. Note that the peptide from EGF2 is not fucosylated. The m/z for each of the fucosylated peptides (top panels) or unmodified peptide (bottom panel, except for EGF2) was used to generate the EIC figures shown in Figure 1B. (PDF) [file pone.0088571.s001.pdf]
